# Supplementary material for: The association between multimorbidity and osteoporosis investigation and treatment in high-risk fracture patients in Australia: A prospective cohort study
Source: PLoS Med. 2023 Jan 17;20(1):e1004142. doi: 10.1371/journal.pmed.1004142 (PMC9844893; doi:10.1371/journal.pmed.1004142)
Supplement: S1 Table — (DOCX) [file pmed.1004142.s002.docx]

**S1 Table List of ICD-10 and ATC coded used to define comorbidities**

| **Comorbidities** | **ICD-10 codes** | **ATC codes**[**^1^**](#_ENREF_1) |
| --- | --- | --- |
| Myocardial infarction^2^ | I21.x, I22.x, I25.2 |  |
| Congestive heart failure | I09.9, I11.0, I13.0, I13.2, I25.5, I42.0, I42.5 - I42.9, I43.x, I50.x, P29.0 | C03DA02–C03DA99, C07AB02, C03CA01– C03CC01 and C09AA01–C09AX99, C09CA01– C09CX99) |
| Peripheral vascular disease | I70.x, I71.x, I73.1, I73.8, I73.9, I77.1, I79.0, I79.2, K55.1, K55.8, K55.9, Z95.8, Z95.9 |  |
| Cerebrovascular disease | G45.x, G46.x, H34.0, I60.x - I69.x |  |
| Dementia | F00.x - F03.x, F05.1, G30.x, G31.1 | N06DA02–N06DA04, N06DX0 |
| Chronic pulmonary disease | I27.8, I27.9, J40.x - J47.x, J60.x - J67.x, J68.4, J70.1, J70.3 | R03AC02–R03DC03, R03DX05 |
| Peptic ulcer disease | K25.x - K28.x | A02BA01–A02BX05 |
| Mild liver disease | B18.x, K70.0 - K70.3, K70.9, K71.3 - K71.5, K71.7, K73.x, K74.x, K76.0, K76.2 - K76.4, K76.8, K76.9, Z94.4 |  |
| Moderate or severe liver disease | I85.0, I85.9, I86.4, I98.2, K70.4, K71.1, K72.1, K72.9, K76.5, K76.6, K76.7 |  |
| Diabetes without chronic complication | E10.0, E10.1, E10.6, E10.8, E10.9, E11.0, E11.1, E11.6, E11.8, E11.9, E12.0, E12.1, E12.6, E12.8, E12.9, E13.0, E13.1, E13.6, E13.8, E13.9, E14.0, E14.1, E14.6, E14.8, E14.9 | A10AA01–A10BX99 |
| Diabetes with chronic complication | E10.2 - E10.5, E10.7, E11.2 - E11.5, E11.7, E12.2 - E12.5, E12.7, E13.2 - E13.5, E13.7, E14.2 - E14.5, E14.7 |  |
| Renal disease | I12.0, I13.1, N03.2 - N03.7, N05.2 - N05.7, N18.x, N19.x, N25.0, Z49.0 - Z49.2, Z94.0, Z99.2 | B03XA01–B03XA03, A11CC01–A11CC04, V03AE02, V03AE03, V03AE05 |
| Any malignancy, including lymphoma and leukaemia, except malignant neoplasm of skin^3^ | C00.x - C26.x, C30.x - C34.x, C37.x - C41.x, C43.x, C45.x - C58.x, C60.x - C76.x, C81.x - C85.x, C88.x, C90.x - C97.x | L01AA01–L01XX41 |
| Metastatic solid tumour | C77.x - C80.x |  |
| AIDS/HIV | B20.x - B22.x, B24.x | J05AE01–J05AE10, J05AF12–J05AG05, J05AR01–J05AR99, J05AX07–J05AX09, J05AX12, J05AF01–J05AF07, J05AF09 |
| Cardiac arrhythmias | I44.1 - I44.3, I45.6, I45.9, I47.x - I49.x, R00.0, R00.1, R00.8, T82.1, Z45.0, Z95.0 | C01AA05, C01BA01–C01BD01, C07AA07 |
| Valvular disease | A52.0, I05.x - I08.x, I09.1, I09.8, I34.x - I39.x, Q23.0 - Q23.3, Z95.2 - Z95.4 |  |
| Hypertension, uncomplicated | I10.x, I11.x - I13.x, I15.x | C03AA01–C03BA11, C03DB01, C03DB99, C03EA01,  C09BA02–C09BA09, C09DA02– C09DA08, C02AB01–C02AC05,  C02DB02– C02DB99 (C03CA01–C03CCO1 or C09CA01– C09CX99) |
