# Supplementary material for: The association between multimorbidity and osteoporosis investigation and treatment in high-risk fracture patients in Australia: A prospective cohort study
Source: PLoS Med. 2023 Jan 17;20(1):e1004142. doi: 10.1371/journal.pmed.1004142 (PMC9844893; doi:10.1371/journal.pmed.1004142)
Supplement: S2 Table — (DOCX) [file pmed.1004142.s003.docx]

S2 Table Clinical risk factors associated with DXA investigation following index hip and vertebral fracture regardless of 10-year Garvan Fracture Risk estimate

|  | Women | | Men | |
| --- | --- | --- | --- | --- |
|  | Age-adjusted  OR (95 % CI) | Multivariable  OR (95%CI) | Age-adjusted  OR (95% CI) | Multivariable  OR (95% CI) |
| Age + 5 years | 0.74 (0.70 - 0.77) | 0.75 (0.71 - 0.79) | 0.85 (0.81 - 0.89) | 0.88 (0.84 - 0.93) |
| Number of comorbidities |  |  |  |  |
| 0 | Reference | Reference | Reference | Reference |
| 1 and 2 | 0.94 (0.68 - 1.30) | 0.96 (0.69 - 1.36) | 1.96 (1.26 - 3.07) | 1.99 (1.26 - 3.16) |
| ≥ 3 | 0.95 (0.68 - 1.33) | 1.06 (0.75 - 1.51) | 1.99 (1.25 - 3.16) | 2.05 (1.27 - 3.30) |
| Number of prior hospitalisations |  |  |  |  |
| 0 | Reference | Reference | Reference | Reference |
| 1 and 2 | 0.68 (0.53 - 0.86) | 0.71 (0.56 - 0.91) | 0.70 (0.53 - 0.92) | 0.68 (0.52 - 0.90) |
| ≥3 | 0.30 (0.15 - 0.58) | 0.36 (0.19 - 0.71) | 0.34 (0.20 - 0.59) | 0.34 (0.20 - 0.60) |
| Charlson Comorbidity Index |  |  |  |  |
| 1 | Reference | Reference | Reference | Reference |
| 2 and 3 | 0.61 (0.47 - 0.78) | 0.65 (0.50 - 0.85) | 0.60 (0.45 - 0.80) | 0.59 (0.44 - 0.79) |
| ≥ 4 | 0.28 (0.17 - 0.45) | 0.29 (0.18 - 0.49) | 0.40 (0.26 - 0.63) | 0.40 (0.25 - 0.63) |
| Ischaemic heart disease | 1.76 (1.27 - 2.44) | 1.84 (1.28 - 2.65) | 1.19 (0.81 - 1.76) | 1.14 (0.76 - 1.71) |
| Arrhythmias | 0.94 (0.66 - 1.36) | 0.75 (0.49 - 1.16) | 0.94 (0.66 - 1.36) | 0.93 (0.64 - 1.35) |
| Stroke | 0.81 (0.50 - 1.30) | 1.11 (0.59 - 2.08) | 0.60 (0.35 - 1.04) | 0.56 (0.30 - 1.04) |
| Diabetes | 0.33 (0.16 - 0.67) | 0.40 (0.19 - 0.84) | 1.00 (0.58 - 1.72) | 0.91 (0.51 - 1.63) |
| Respiratory disease | 0.83 (0.63 - 1.11) | 0.98 (0.72 - 1.35) | 1.06 (0.76 - 1.47) | 1.05 (0.75 - 1.48) |
| Renal disease | 0.56 (0.28 - 1.11) | 0.49 (0.23 - 1.04) | 0.46 (0.22 - 0.95) | 0.48 (0.23 - 0.99) |
| Dementia | 0.04 (0.005 - 0.26) | 0.04 (0.005 - 0.29) | 0.17 (0.05 - 0.53) | 0.20 (0.06 - 0.64) |
| Cancer | 1.04 (0.81 - 1.34) | 1.13 (0.86 - 1.50) | 1.09 (0.83 - 1.42) | 1.06 (0.80 - 1.40) |
| Peptic ulcer | 0.99 (0.71 - 1.39) | 1.07 (0.78 - 1.46) | 1.61 (1.17 - 2.20) | 0.94 (0.65 - 1.34) |
| Aged care residency | 0.54 (0.28 - 1.05) | 0.65 (0.32 - 1.31) | 0.62 (0.29 - 1.30) | 0.57 (0.26 - 1.27) |
| Disability | 0.49 (0.34 - 0.70) | 0.51 (0.32 - 1.31) | 0.80 (0.54 - 1.20) | 0.82 (0.53 - 1.26) |
| Smoking | 0.79 (0.52 - 1.20) | 0.87 (0.55 - 1.38) | 1.21 (0.80 - 1.83) | 1.28 (0.83 - 1.97) |
| Private health insurance | 1.49 (1.20 - 1.84) | 1.48 (1.16 - 1.89) | 1.26 (0.99 - 1.61) | 1.20 (0.93 - 1.56) |
| Married | 1.37 (1.10 - 1.69) | 1.43 (1.13 - 1.81) | 0.95 (0.74 - 1.24) | 1.10 (0.84 - 1.44) |
