# Supplementary material for: The association between multimorbidity and osteoporosis investigation and treatment in high-risk fracture patients in Australia: A prospective cohort study
Source: PLoS Med. 2023 Jan 17;20(1):e1004142. doi: 10.1371/journal.pmed.1004142 (PMC9844893; doi:10.1371/journal.pmed.1004142)
Supplement: S3 Table — (DOCX) [file pmed.1004142.s004.docx]

S3 Table Clinical risk factors associated with treatment investigation following index hip and vertebral fracture regardless of 10-year Garvan Fracture Risk estimate

|  | Women | | Men | |
| --- | --- | --- | --- | --- |
|  | Age-adjusted  OR (95 % CI) | Multivariable  OR (95%CI) | Age-adjusted  OR (95% CI) | Multivariable  OR (95% CI) |
| Age + 5 years | 0.94 (0.90 - 0.98) | 0.96 (0.91 - 1.00) | 1.00 (0.95 - 1.05) | 1.51 (0.98 - 2.33) |
| Number of comorbidities |  |  |  |  |
| 0 | Reference | Reference | Reference | Reference |
| 1 and 2 | 0.85 (0.62 - 1.16) | 0.85 (0.62 - 1.17) | 1.59 (1.04 - 2.44) | 1.51 (0.98 - 2.34) |
| ≥ 3 | 0.84 (0.61 - 1.15) | 0.83 (0.60 - 1.17) | 1.45 (0.94 - 2.25) | 1.29 (0.82 - 2.01) |
| Number of prior hospitalisations |  |  |  |  |
| 0 | Reference | Reference | Reference | Reference |
| 1 and 2 | 0.90 (0.73 - 1.12) | 0.90 (0.72 - 1.12) | 0.96 (0.75 - 1.23) | 0.97 (0.75 - 1.25) |
| ≥3 | 0.54 (0.33 - 0.88) | 0.56 (0.34 - 0.93) | 0.49 (0.31 - 0.78) | 0.46 (0.29 - 0.73) |
| Charlson Comorbidity Index |  |  |  |  |
| 1 | Reference | Reference | Reference | Reference |
| 2 and 3 | 0.79 (0.63 - 0.99) | 0.79 (0.62 - 0.99) | 0.90 (0.89 -1.46) | 1.08 (0.92 - 1.53) |
| ≥ 4 | 0.69 (0.48 - 0.99) | 0.70 (0.48 - 1.00) | 0.49 (0.33 - 0.74) | 0.46 (0.30 - 0.71) |
| Ischaemic heart disease | 1.15 (0.84 - 1.58) | 1.07 (0.78 - 1.48) | 0.84 (0.56 - 1.24) | 0.86 (0.57 - 1.30) |
| Arrhythmias | 0.78 (0.56 - 1.09) | 0.81 (0.57 - 1.14) | 1.06 (0.78 - 1.45) | 1.05 (0.76 - 1.46) |
| Stroke | 0.77 (0.51 - 1.17) | 1.08 (0.65 - 1.82) | 0.72 (0.46 - 1.13) | 0.67 (0.39 - 1.13) |
| Diabetes | 0.53 (0.30 - 0.93) | 0.54 (0.30 - 0.95) | 0.88 (0.52 - 1.49) | 0.90 (0.55 - 1.52) |
| Respiratory disease | 0.89 (0.68 - 1.16) | 0.88 (0.67 - 1.17) | 1.11 (0.81 - 1.50) | 1.10 (0.80 - 1.52) |
| Renal disease | 0.77 (0.44 - 1.35) | 0.86 (0.48 - 1.52) | 0.48 (0.25 - 0.90) | 0.49 (0.26 - 0.92) |
| Dementia | 0.38 (0.22 - 0.66) | 0.38 (0.22 - 0.66) | 0.64 (0.37 - 1.12) | 0.78 (0.44 - 1.37) |
| Cancer | 0.85 (0.67 - 1.08) | 0.83 (0.65 - 1.06) | 1.30 (1.02 - 1.65) | 1.24 (0.97 - 1.60) |
| Peptic ulcer | 0.81 (0.62 - 1.06) | 0.78 (0.59 - 1.03) | 1.38 (1.03 - 1.85) | 1.34 (0.90 - 1.82) |
| Aged care residency | 0.52 (0.29 - 0.92) | 0.58 (0.32 - 1.05) | 0.69 (0.37 - 1.32) | 0.77 (0.40 - 1.48) |
| Disability | 0.99 (0.75 - 1.31) | 1.15 (0.85 - 1.53) | 1.26 (0.91 - 1.76) | 1.28 (0.90 - 1.82) |
| Smoking | 1.29 (0.88 - 1.89) | 1.36 (0.91- 2.02) | 0.78 (0.49 - 1.25) | 0.82 (0.50 - 1.34) |
| Private health insurance | 1.18 (0.98 - 1.44) | 1.18 (0.96 - 1.45) | 1.25 (0.99 - 1.56) | 1.29 (1.02 - 1.65) |
| Married | 1.14 (0.94 - 1.40) | 1.10 (0.89 - 1.35) | 0.98 (0.77 - 1.25) | 1.11 (0.86 - 1.44) |
