# Supplementary material for: The association between multimorbidity and osteoporosis investigation and treatment in high-risk fracture patients in Australia: A prospective cohort study
Source: PLoS Med. 2023 Jan 17;20(1):e1004142. doi: 10.1371/journal.pmed.1004142 (PMC9844893; doi:10.1371/journal.pmed.1004142)
Supplement: S4 Table — (DOCX) [file pmed.1004142.s005.docx]

S4 Table Clinical risk factors associated with DXA investigation in all participants regardless of treatment initiation

|  | Women | | Men | |
| --- | --- | --- | --- | --- |
|  | Age-adjusted OR | Multivariable OR | Age-adjusted OR | Multivariable OR |
| Age + 5 years | 0.78 (0.76 - 0.80) | 0.79 (0.77 - 0.82) | 0.48 (0.41 - 0.55) | 0.47 (0.41 - 0.55) |
| Number of comorbidities |  |  |  |  |
| 0 | Reference | Reference | Reference | Reference |
| 1 and 2 | 1.05 (0.92 - 1.21) | 1.09 (0.94 - 1.25) | 1.14 (0.69 - 1.90) | 1.14 (0.67 - 1.92) |
| ≥ 3 | 1.09 (0.94 - 1.26) | 1.15 (0.99 - 1.33) | 1.01 (0.61 - 1.67) | 1.02 (0.67 - 1.92) |
| Number of prior hospitalisations |  |  |  |  |
| 0 | Reference | Reference | Reference | Reference |
| 1 and 2 | 0.78 (0.69 - 0.88) | 0.80 (0.71 - 0.91) | 0.58 (0.45 - 0.75) | 0.61 (0.47 - 0.79) |
| ≥3 | 0.46 (0.33 - 0.63) | 0.49 (0.35 - 0.67) | 0.41 (0.26 - 0.63) | 0.43 (0.27 - 0.67) |
| Charlson Comorbidity Index |  |  |  |  |
| 1 | Reference | Reference | Reference | Reference |
| 2 and 3 | 0.71 (0.62 - 0.81) | 0.73 (0.64 - 0.84) | 0.45 (0.35 - 0.59) | 0.46 (0.35 - 0.61) |
| ≥ 4 | 0.49 (0.38 - 0.62) | 0.51 (0.40 - 0.65) | 0.36 (0.24 - 0.54) | 0.38 (0.25 - 0.57) |
| Ischaemic heart disease | 1.34 (1.14 - 1.57) | 1.40 (1.17 - 1.68) | 1.42 (1.02 - 1.97) | 1.42 (1.01 - 1.98) |
| Arrhythmias | 0.75 (0.62 - 0.92) | 0.75 (0.60 - 0.93) | 0.77 (0.56 - 1.05) | 0.74 (0.54 - 1.03) |
| Stroke | 0.91 (0.70 - 1.18) | 0.94 (0.68 - 1.30) | 0.61 (0.39 - 0.97) | 0.63 (0.38 - 1.05) |
| Diabetes | 0.50 (0.37 - 0.68) | 0.57 (0.41 - 0.80) | 0.75 (0.42 - 1.34) | 0.74 (0.31 - 1.32) |
| Respiratory disease | 0.99 (0.88 - 1.12) | 1.08 (0.94 - 1.25) | 1.07 (0.79 - 1.46) | 1.15 (0.84 - 1.58) |
| Renal disease | 0.60 (0.40 - 0.90) | 0.58 (0.37 - 0.91) | 0.61 (0.34 - 1.09) | 0.56 (0.31 - 1.04) |
| Dementia | 0.20 (0.11 - 0.36) | 0.22 (0.12 - 0.39) | 0.31 (0.14 - 0.67) | 0.33 (0.14 - 0.76) |
| Cancer | 1.07 (0.95 - 1.21) | 1.17 (1.02 - 1.34) | 0.95 (0.74 - 1.21) | 0.91 (0.70 - 1.17) |
| Peptic ulcer | 1.14 (1.01 - 1.30) | 1.17 (1.01 - 1.35) | 0.79 (0.56 - 1.12) | 0.82 (0.58 - 1.17) |
| Aged care residency | 0.62 (0.44 - 0.86) | 0.57 (0.40 - 0.92) | 0.89 (0.42 - 1.88) | 0.96 (0.43 - 2.15) |
| Disability | 0.74 (0.62 - 0.89) | 0.76 (0.62 - 0.92) | 0.55 (0.35 - 0.87) | 0.63 (0.39 - 1.02) |
| Smoking | 0.71 (0.57 - 0.89) | 0.68 (0.54 - 0.86) | 0.58 (0.28 - 1.18) | 0.55 (0.25 - 1.17) |
| Private health insurance | 1.23 (1.11 - 1.36) | 1.33 (1.18 - 1.49) | 1.52 (1.20 - 1.93) | 1.49 (1.17 - 1.90) |
| Married | 1.12 (1.01 - 1.23) | 1.13 (1.01 - 1.26) | 0.83 (0.65 - 1.07) | 0.80 (0.62 - 1.04) |
