# Supplementary material for: The association between multimorbidity and osteoporosis investigation and treatment in high-risk fracture patients in Australia: A prospective cohort study
Source: PLoS Med. 2023 Jan 17;20(1):e1004142. doi: 10.1371/journal.pmed.1004142 (PMC9844893; doi:10.1371/journal.pmed.1004142)
Supplement: Attachment — Submitted filename: bliue.pdf [file pmed.1004142.s006.pdf]

This paper links various administrative databases to study the association in patients at high risk of fracture between multimorbidity and their subsequent fate in the health service.

I was asked for a statistical report and I interpret that to include all aspects of the design and conduct of the study.

Points of detail

Page 4 Why randomly sample? Surely issues like the cost of data collection do not apply here and a larger sample would be essentially free. An explicit statement about the designed analytical sample size might be a good idea.

Page 5 I think it might be helpful to spell out what NSW is for an international readership. Perhaps more importantly the abstract tells us this study was on people in Australia but this section seems to restrict it to NSW. Is that true?

Page 5 Why were certain fractures excluded? How was 'high trauma' defined and who recorded it?

Page 5 Why was the CCI categorised like this? It seems to have ended up with about three quarters of the sample in the bottom category which hardly seems optimal. A glossary of terms might be a good idea too. I know it was spelled out earlier but it is awkward to have to look back to find it (for readers who print articles).

Page 6 The paragraph starting 'Sixty two percent' is, strictly speaking part of the results.

Page 6 Presumably 'men are less likely ... ' applies to this condition. I do not think it is universal in health care.

Page 7 Given the substantial differences in fracture site between men and women I am surprised that it was not included as an adjustment variable in the models especially when sex differences are being investigated. For instance about 51% of the women but only 38% of the men sustained a distal fracture (using the DXA columns, the Rx columns have very different N). Indeed the results section shows the authors have noted this difference.

Page 7 If the cohort was arrived at by probability sampling as page 4 states then it needs to be taken into account in the analysis but I do not see it mentioned here or later in the results.

Page 7 What exactly does the ± notation mean? If this is a confidence interval then I am surprised that it is symmetrical given the proximity of the ages to the practical upper bound.

Page 7 What does $27 - 53\%$ mean? Is this a confidence interval and if so how was it calculated and what is the estimate about which it lies? If it is not a confidence interval then what is it?

Page 8 If there is a clear sex difference then we need to see the values for each sex separately and not just, as on the previous page, an overall value which applies to neither sex.

Page 8 How were the figures for the numbers prevented arrived at and what model was used to determine the confidence intervals quoted here? This is not spelled out in the methods as far as I can see.

Page 10 What is BMD? If it is bone mineral density, as seems possible, how plausible is it to use weight as a surrogate for it?

Summary

The whole article is rather short of detail especially on why the authors did what they did. The non–use of survey weights and the apparent disregard of fracture site need attention.

Michael Dewey
