# Supplementary material for: The association between multimorbidity and osteoporosis investigation and treatment in high-risk fracture patients in Australia: A prospective cohort study
Source: PLoS Med. 2023 Jan 17;20(1):e1004142. doi: 10.1371/journal.pmed.1004142 (PMC9844893; doi:10.1371/journal.pmed.1004142)
Supplement: Attachment — Submitted filename: Rebuttal.4Oct.22.docx [file pmed.1004142.s007.docx]

29 September 2022

Dr Callam Davidson,
Associate Editor

PLOS Medicine
plosmedicine.org

**Re: Manuscript PMEDICINE-D-22-02127R1**

Dear Dr Davidson,

We thank the editors and the reviewers for the extensive review of our manuscript and for their positive appraisal of our work. We have carefully considered the comments and made all the changes as requested.

Specifically, we have changed the title and have included a series of sensitivity analyses of the whole cohort as well as restricted to hip and vertebral fractures which are considered the most severe fracture sites. The consistency between the results from the sensitivity and the primary analyses, emphasize the robustness of our findings.

We hope that these revisions answer your concerns and that it will now be acceptable for publication in *PLOS MEDICINE*.

Yours sincerely,

Dr. Dana Bliuc

On behalf of all authors

**Manuscript PMEDICINE-D-22-02127R1**

We addressed every point raised with a response and show, in red, modified manuscript text.

**Comment**: “Comments from the Academic Editor:
1/ The rationale may have been set in part by convenience based on the available dataset”

**Answer:** It is well known that fractures are commonly associated with multiple chronic conditions. Currently osteoporosis guidelines are based on a “single-disease” approach, due to the lack of information on the effectiveness of osteoporosis medication in sicker patients. We have obtained a grant to investigate the effectiveness of osteoporosis medication in people with multimorbidity. The current manuscript was developed to investigate to what degree the presence of other chronic conditions affects the uptake of medication to prevent future fractures.

The 45 and Up Study cohort, a large population-based study including detailed questionnaires linked to administrative health databases including hospital admissions, Medicare claims, prescriptions databases provided the level of information needed to determine the relationship between osteoporosis investigation and treatment uptake in people with multiple chronic conditions and at high-risk of fracture.

We have articulated better our study rationale and hypothesis in the Introduction:

Furthermore, the role of major comorbidities on fracture prevention management is unknown. We hypothesised that multimorbidity, defined as number and severity of chronic conditions present at the time of osteoporotic fracture, decreases the rate of osteoporosis investigation and treatment. We studied this in people with an index minimal trauma fracture and high-risk of subsequent fracture in a well-characterized large population-based study.

We also emphasize the strength of the study in Discussion:

This study’s strengths include thorough documentation of chronic conditions from three different sources (self-report, hospital admissions, and prescription databases) and osteoporosis investigation (from Medicare claims) and treatment (prescription databases) in a large population-based cohort[1].

**Comment*:*** *“2/ It is unclear how reliable the classification of patients with and without comorbidities is”*

**Answer:** Thank you for the comment. In the current study we have used validated algorithms for the identification of chronic comorbidities to minimise classification error. To identify multimorbidity, we have used Charlson Comorbidity Index (CCI) with proven high positive predictive value in multiple studies. In Danish National Registry of Patients, the overall positive predictive value for CCI conditions from ICD10 codes was 98.0 % (95% CI, 96.9 – 98.8) for ICD10 codes compared with the diagnosis obtained from discharge records[2]. Given this high concordance between information recorded by health professionals and that entered in administrative registries reported in previous studies, we believe that the classification of multimorbidity using CCI in our study is very reliable.

In addition to the CCI scores we also examined separately all the conditions included in the CCI score and those additional conditions captured by questionnaire (ie hypertension, rheumatoid arthritis, Parkinson disease) if their prevalence ≥ 3%. Individual chronic conditions identification was based on APDC, questionnaires, and PBS datasets. As mentioned above, we are confident that the classification of comorbidities based on ICD10 codes from APDC dataset is very reliable. However, self-reported information from questionnaire is subject to recall bias. The classification of comorbidities using the RxRisk medication-based algorithm[1, 2] is based on the concept that each comorbidity has a specific pharmacological treatment. A limitation of the RxRisk model is that comorbidities without a specific medication would be underdiagnosed. In order to minimise these limitations, we have used the combination of multiple data sources as described.

We have added more details on multimorbidity definition and sources in the Methods section.

**Multimorbidity definition and identification**

Multimorbidity was determined using Charlson Comorbidity Index (CCI). In addition, individual comorbidities either included in CCI or self-reported in questionnaire (i.e. hypertension, rheumatoid arthritis, Parkinson disease, anxiety, depression) were also identified.

CCI was estimated from hospital admissions dataset (APDC) and was based on ICD-10 codes using a 5-year look back window prior to index fracture. Chronic conditions included in CCI were identified from all 50 hospital diagnosis codes available. Previous studies have reported high positive predictive value for CCI obtained from administrative registries[2]. A full list of the codes or self-report diagnoses used to identify chronic conditions is provided in a supplemental table (Table S1). CCI was classified in 3 groups: low morbidity CCI ≤1 (referent group), mild morbidity 2-3, and severe morbidity ≥4. These groups were chosen to discriminate between those with small comorbidity burden (up to 1 chronic condition), mild morbidity (2-3 chronic conditions) and severe morbidity (more than 4 chronic conditions).

We have also explored multimorbidity as number of comorbidities, and number of hospitalisations within 5-years prior to index fracture.

In addition to CCI and the unweighted count of conditions, specific conditions with a prevalence ≥3% were analysed and reported separately. These specific chronic conditions were identified using three data sources: self-report, hospital admissions and prescription databases cumulatively for up to 5 years prior to index fracture. In the questionnaires, participants were asked whether “a doctor has ever told you that you have a chronic condition (yes/no)”. The ATC codes in the prescription database (PBS) were used to derive information about chronic conditions using the Rx-Risk Index[1][23][22]. The Rx-Risk model mapped up to 46 conditions using ATC medication codes from PBS (Table S1).

We have also acknowledged the limitation in the classification of individual chronic conditions.

Self-reported comorbidities may be subjected to recall bias, and classification of comorbidities based on prescription would possibly underdiagnose conditions without a specific pharmacological medication. In order to account for these limitations we have used multiple data sources for comorbidities identification, a strategy that has been proven to be the best for comorbidities classification[3].

**Comment*:*** *“3/ Reporting is lacking important information, especially in the results section where p values are presented alone, without the results.”*

**Answer:** Thank you for the comment. We have revised the manuscript accordingly. Changes are too scattered to be worth noting individually here.

**Comment*:*** *“Requests from the editors:
Please update your title to ‘The association of multimorbidity and osteoporosis investigation and treatment in high-risk fracture patients in Australia: a cohort study’.”*

**Answer:** Thank you for the comment. We have changed the title to *‘*Multimorbidity is associated with lower chance of osteoporosis investigation and treatment in high-risk fracture patients in Australia: a prospective cohort study’

**Comment*:*** *“Please structure your abstract using the PLOS Medicine headings (Background, Methods and Findings, Conclusions).”*

**Answer:** Thank you for the comment. We have re-structured the Abstract according to PLOS Medicine headings.

**Comment*:*** *“Abstract Background: Provide the context of why the study is important. The final sentence should clearly state the study question.”*

**Answer:** We have now added the importance of our research question in the Abstract Background.

**Background:** Multimorbidity is common among fracture patients. However, its association with osteoporosis investigation and treatment to prevent future fractures is unclear. This limited knowledge impedes optimal patient care.

**Comment*:*** *“Your study is observational and therefore causality cannot be inferred. Please remove language that implies causality, such as ‘impact’ and ‘contribution’. Refer to associations instead.”*

**Answer:** We agree with your comment. We have now replaced all the words implying causality with “associations” throughout the manuscript.

**Comment*:*** *“Abstract Methods and Findings*

** Please ensure that all numbers presented in the abstract are present and identical to numbers presented in the main manuscript text.*
** Please include the years during which the study took place and length of follow up.*
** Please quantify the main results (with 95% CIs and p values).
* Please include the important dependent variables that are adjusted for in the analyses.
* In the last sentence of the Abstract Methods and Findings section, please describe the main limitation(s) of the study's methodology.”*

**Answer:** Thank you for the comment. We have made all the above changes to the Abstract to fit with PLOS Medicine requirements.

**Background:** Multimorbidity is common among fracture patients. However, its association with osteoporosis investigation and treatment to prevent future fractures is unclear. This limited knowledge impedes optimal patient care. This study investigated the association between multimorbidity and osteoporosis investigation and treatment in persons at high risk of future fracture following an index osteoporotic fracture.

**Methods and findings:** The Sax Institute’s 45 and Up Study is a prospective population-based cohort of 267,153 people in Australia recruited between 2005/2009. This analysis followed up subjects until 2017 for a median of 6 years (IQR: 4-8). Questionnaire data were linked to hospital admissions (Admitted Patients Data Collection –APDC), emergency presentations (Emergency Department Data Collection – EDDC), Pharmaceutical Benefits Scheme (PBS) and Medicare Benefits Schedule (MBS). Data were linked by the Centre for Health Record Linkage and stored in a secured computing environment. Fractures were identified from APDC and EDDC; Charlson Comorbidity Index (CCI) from APDC; DXA investigation from MBS; osteoporosis treatment from PBS.

Out of 25,280 persons with index fracture, 10,540 were classified as high-risk based on 10-year Garvan fracture risk calculator (age, sex, weight, prior fracture and falls) threshold ≥ 20%. The association of CCI with likelihood of investigation and treatment initiation was determined by logistic regression adjusted for education, socio-economic and lifestyle factors. The high-risk women and men averaged 77 ± 10 and 86 ± 5 years, respectively; > 40% had a CCI ≥ 2. Only 17% of women and 7% of men received a DXA referral, and 22% of women and 14% men received osteoporosis medication following fracture. A higher CCI was associated with a lower probability of being investigated [adjusted OR, women: 0.73 (95% CI, 0.61 – 0.87) and 0.43 (95% CI, 0.30 – 0.62); men: 0.47 (95% CI, 0.33 – 0.68) and 0.52 (0.31 – 0.85) for CCI: 2-3, and ≥ 4 vs 0-1, respectively] and of receiving osteoporosis medication [adjusted OR, women: 0.85 (95% CI, 0.74 – 0.98) and 0.78 (95% CI, 0.61 – 0.99); men: 0.75 (95% CI, 0.59 – 0.94) and 0.37 (95% CI, 0.23 – 0.53) for CCI: 2-3, and ≥ 4 vs 0-1, respectively].

**Conclusions:** Multimorbidity was significantly associated with the osteoporosis treatment gap. This suggests that fracture risk is either under-estimated or under-prioritized in the context of multimorbidity and highlights the need for extra vigilance and improved fracture care in this setting.

**Limitation:** The cohort is relatively healthy, therefore the impact of multimorbidity on osteoporosis management may have been underestimated.

**Comment:** *“The Data Availability Statement (DAS) requires revision. If the data are not freely available, please describe briefly the ethical, legal, or contractual restriction that prevents you from sharing it. If the data are available upon request, please note this and state the owner of the data set and contact information for data requests (web or email address). Note that a study author cannot be the contact person for the data.”*

**Answer:** We have updated the Data Availability Statement in accordance with the Sax Institute.

Data cannot be shared publicly as they are owned by the Sax Institute, which is the data custodian for the 45 and Up Study. Anyone wishing to request access can do so through the 45 and Up Study. The data are available for researchers who meet the criteria for access to confidential data. Details are available at <https://www.saxinstitute.org.au/our-work/45-up-study/> and requests to access data can be submitted via email to [45andUp.Research@saxinstitute.org.au](mailto:45andUp.Research@saxinstitute.org.au).

**Comment:** *“Please do not use footnotes (such as those used in the Abstract). Relevant information should be included in the main text.”*

**Answer:** We have included the relevant information in the body of Abstract and removed the footnotes.

**Comment*:*** *“At this stage, we ask that you include a short, non-technical Author Summary of your research to make findings accessible to a wide audience that includes both scientists and non-scientists. The Author Summary should immediately follow the Abstract in your revised manuscript. This text is subject to editorial change and should be distinct from the scientific abstract. Please see our author guidelines for more information:* [*https://journals.plos.org/plosmedicine/s/revising-your-manuscript#loc-author-summary*](https://protect-au.mimecast.com/s/y6rlCp81qMuzyLg8h1byZA?domain=journals.plos.org)*”*

**Answer:** We have included an Author Summary.

**Author Summary:**

**Why was this study done:**

- Osteoporotic fractures are common, costly and associated with increased risk of future fracture and premature mortality
- Effective preventive treatment is available and recommended for high-risk patients with a 10-year fracture risk >20%, but its uptake in this group is suboptimal
- High-risk patients often have multiple chronic conditions
- It is important to know to what degree the other chronic conditions might alter the uptake of fracture prevention medication in order to optimise patient care

**What did the researchers do and find:**

- We conducted a prospective study including over 10,000 women and men aged 50+ with an osteoporotic fracture and high-risk of future fracture
- In this high-risk group, the vast majority of women and men were not investigated or treated for osteoporosis following an osteoporotic fracture
- Women and men with multiple chronic conditions were significantly less likely to be investigated or treated for osteoporosis compared to those without chronic conditions

**What do these findings mean:**

- The presence of additional chronic conditions was associated with a lower likelihood of being investigated and treated for osteoporosis
- These findings suggests that fracture preventing treatment is either under-prioritised or under-estimated in the presence of other chronic conditions
- There is a need for more awareness of increased fracture risk in complex patients with multiple chronic conditions.

**Comment***: “Please update citations to be non-superscript, placed in square brackets, and preceding punctuation.”*

**Answer:** We have updated the references as suggested.

**Comment:** *“The terms gender and sex are not interchangeable (as discussed in* [*https://www.who.int/health-topics/gender*](https://protect-au.mimecast.com/s/uOqnCq71rNfORrg5fzBupP?domain=who.int)*); please use the appropriate term.”*

**Answer:** Thank you for the comment. We have revised the manuscript and replace gender with sex.

**Comment: *“****Please provide a reference for the statement at line 198-199.”*

**Answer:** We have added a reference as suggested.

**Comment:** *“Please correct the typo at line 144. “*

**Answer:** Thank you for the comment. We have corrected the error.
In the questionnaires, participants were asked whether “a doctor has ever told you that you have a chronic condition (yes/no)”.

**Comment:** *“Please specify whether informed consent was written or oral.”*

**Answer:** Participants have provided written consent to participate in the study.

We have made this clear in Methods.

All participants provided signed consent to take part in the study and to have their data linked to administrative datasets.

**Comment: *“****Please define "lost to follow-up" as used in this study. Other reasons for exclusion should be defined.”*

**Answer:** In this study we did not have any lost to follow-up. Both our outcomes are based on linked administrative databases. Investigation for osteoporosis by DXA was identified from Medicare Benefits Schedule (MBS) and treatment for osteoporosis from Pharmaceutical Benefits Scheme (PBS). Every Australian resident receiving these services would have an entry into these databases. Importantly, PBS and MBS are nation-wide databases, meaning that even if participants moved to another state their PBS and MBS data can still be ascertained.

We have added this information to Methods.

The primary outcome measurements included investigation for osteoporosis by DXA and treatment for osteoporosis initiated after the index fracture. Both were identified from nationwide administrative databases, ensuring a complete ascertainment for all participants.

**Comment:** *“Please remove the ‘Disclosures’ section from the end of the manuscript and ensure all relevant information is captured in the ‘Competing Interests’ section of the submission form. “*

**Answer:** Thank you for your comment. We have removed the Disclosures section from the manuscript and included this information in the “Competing Interests” section of the submission form.

**Comment:** *“Please use et al only after listing the first six authors in your References.”*

**Answer:** Thank you for your comment. We have updated the References to include up to 6 authors.

**Comment: *“****Table 1: Please define Rx in the legend.”*

**Answer:** We have included a definition of Rx in the footnote of Table 1.
The following sentence was added to Table 1:

1-Treatement included at least one prescription of bisphosphonates, denosumab, teriparatide, strontium ranelate, selective estrogen receptor modulators (SERMS) and hormone therapy initiated at any time following the index fracture

**Comment: *“****Please ensure that the study is reported according to the STROBE guideline, and include the completed STROBE checklist as Supporting Information. Please add the following statement, or similar, to the Methods: "This study is reported as per the Strengthening the Reporting of Observational Studies in Epidemiology (STROBE) guideline (S1 Checklist)."

The STROBE guideline can be found here:* [*http://www.equator-network.org/reporting-guidelines/strobe/*](https://protect-au.mimecast.com/s/wkGECr81vOuA6jyviP9xVE?domain=equator-network.org) *When completing the checklist, please use section and paragraph numbers, rather than page numbers.”*

**Answer:** Thank you for your comment. We have formatted the manuscript according to STROBE guidelines. We have also provided the STROBE checklist as S1 Checklist, indicating the lines in the manuscript for each point.

**Comment:** “*Did your study have a prospective protocol or analysis plan? Please state this (either way) early in the Methods section.

a) If a prospective analysis plan (from your funding proposal, IRB or other ethics committee submission, study protocol, or other planning document written before analyzing the data) was used in designing the study, please include the relevant prospectively written document with your revised manuscript as a Supporting Information file to be published alongside your study, and cite it in the Methods section. A legend for this file should be included at the end of your manuscript.
b) If no such document exists, please make sure that the Methods section transparently describes when analyses were planned, and when/why any data-driven changes to analyses took place.
c) In either case, changes in the analysis-- including those made in response to peer review comments-- should be identified as such in the Methods section of the paper, with rationale.”*

**Answer:** The current study was developed following a successful grant application proposal to investigate the effectiveness of osteoporosis medication in people with multiple chronic conditions. This proposal involved building of a “whole of population” linked study to include sicker participants who are usually excluded from randomised controlled trials or prospective cohorts. The current analysis was based in part on the grant proposal and aimed to investigate the magnitude of association between multimorbidity and osteoporosis treatment uptake. The definitions for the high fracture risk groups, multimorbidity and treatment groups were based on the grant proposal. Notably, treatment groups in the current manuscript included also hormone therapy, which was not included in the grant proposal. All the analyses included in this manuscript were discussed with the senior authors prior to study commencement. There were no analysis changes made based on findings. The sensitivity analyses and the risk of subsequent fracture and mortality were added during the revision process.

We have added the following statement to Methods:

This is a prospective study involving a large population-based study involving questionnaire data linked to administrative health databases. This study was developed as a part of a successful funding proposal (2020 Amgen International Competitive Bone Grant ProliaBCGP-06) to determine the magnitude of association between multimorbidity and osteoporosis treatment gap in patients at high risk of fracture. The definition for each of the high-risk groups, multimorbidity and treatment groups were predefined prior to the commencement of data analysis, as were the details of the prevented fracture estimation. The sensitivity analyses and the risk of subsequent fracture and mortality were added during the revision process.

**REVIEWER # 1**

**Comment: *“****This paper links various administrative databases to study the association in patients at high risk of fracture between multimorbidity and their subsequent fate in the health service. I was asked for a statistical report and I interpret that to include all aspects of the design and conduct of the study.*

*Points of detail*

*Page 4 Why randomly sample? Surely issues like the cost of data collection do not apply here and a larger sample would be essentially free. An explicit statement about the designed analytical sample size might be a good idea.”*

**Answer:** Thank you for the comment. The current study is a prospective population-based study run by the Sax Institute in NSW, Australia. The study involved participant recruitment with questionnaire administration and data linkage to administrative databases. The 45 and Up study was developed to provide evidence for a large range of health-related outcomes relevant for healthy ageing. Individuals 45 and over were randomly selected from Medicare Enrolment Database, the national healthcare provider with almost complete coverage for all Australian permanent residents and citizens. The study was oversampled for the group 85+ and those living in rural areas to allow for death and loss to follow-up. The study aimed to recruit ~ 10.9% of the target general population. From the total 267,135 participants enrolled in the 45 and Up Study, we have included all persons who sustained an index fracture between enrolment 2005/2009 and 2016.

We have made this clear in Methods.

This study included women and men aged 45 and over enrolled in the Sax Institute 45 and Up Study with a confirmed index fracture. The 45 and Up Study is an ongoing prospective cohort study that aims to characterise population health and ageing in New South Wales, Australia through detailed questionnaires and linkage of all available administrative health databases. A detailed description of the study design and recruitment has been published previously[4, 5]. Participants were randomly sampled from the Services Australia (formerly the Australian Government Department of Human Services) Medicare enrolment database, which provides near complete coverage of the population. The target population comprised women and men over 45 years of age with oversampling of the rural population and individuals aged over 80, to account for death and loss of follow-up. Recruitment took place between 2005 and 2009 and the response rate was 18%.

A description of the selection of analytical sample was included in the Results.

Of the total 267,153 participants (266, 921 with accurate records) enrolled in the 45 and Up cohort, 25,280 (16,191 women and 9089 men) sustained an index osteoporotic fracture. Of the total individuals with index fracture, 15,083 (60%) were classified as being at high-risk of subsequent fracture based on a 10-year Garvan Fracture Risk Calculator estimate ≥ 20% (Figure 1). More women (76%) than men (61%) were classified into the high-risk group.

Compared to the low-risk group, in the high-risk group, individuals were older [age, years mean (SD), women: 77 (10) vs 57 (4) and men: 86 (5) vs 65 (8), for high vs low-risk groups, respectively], and had more severe fractures [index hip and vertebral fracture n (%), women: 3051 (26%) vs 223 (6%) and men: 1500 (45%) vs 981 (18%) for high vs low risk groups, respectively]. Those in the high-risk group also had a higher CCI [women, (≤ 1, 2 and 3, ≥ 4) was 70%, 24%, 7% for high-risk and 88%, 9%, 3% for low-risk (p-value <0.0001); men 47%, 38%, 15% for high, and 75%, 18%, 7% for low-risk (p-value <0.0005)] and a higher prevalence of cardio-vascular, diabetes, respiratory, renal disease, cancer and dementia. There was no difference in the level of education between the two fracture risk groups.

A further 4543 individuals were already on osteoporosis treatment and excluded from analysis.

The analytical cohort consisted of 10,540 women and men with a 10-year risk of subsequent fracture ≥ 20% who were not on treatment at the time of index fracture.

**Comment: *“****Page 5 I think it might be helpful to spell out what NSW is for an international readership. Perhaps more importantly the abstract tells us this study was on people in Australia but this section seems to restrict it to NSW. Is that true?”*

**Answer:** Thank you for the comment. The study involved New South Wales residents enrolled in the 45 and Up Study.

We have made this clear in Abstract and Methods.

The study population consisted of 10,540 women and men enrolled in the Sax Institute 45 and Up Study with a confirmed index fracture. The 45 and Up Study is an ongoing prospective cohort study that aims to characterise population health and ageing in New South Wales (NSW), Australia through detailed questionnaires and linkage of all available administrative health databases.

**Comment:** *“Page 5 Why were certain fractures excluded? How was ‘high trauma’ defined and who recorded it?”*

**Answer:** Thank you for the comment. In our analysis, we focused on fracture that would be universally accepted as being “osteoporotic”. The most common definition of osteoporotic fracture is based on the condition of occurrence as a result of a minimal trauma fracture. For this reason, we have excluded ‘high-trauma’ fractures because they could be perceived by clinicians and patients as being ‘non-osteoporotic”. We have defined ‘high-trauma” using ICD10 codes. In addition, we have excluded the fracture events that resulted in more than 4 fracture sites occurring concomitantly. This criterion was based on the clinicians’ experience that simultaneous fracture at more than 4 sites likely involves significant trauma.

We have made this clear in Methods.

High trauma fractures as well as fractures of digits, skull and face were excluded from the analysis as they are commonly considered “non-osteoporotic”. High trauma fractures were identified from ICD-10 codes. In addition, the occurrence of more than 4 fractures at different sites during the same event was considered likely the result of high trauma and were excluded.

**Comment: *“****Page 5 Why was the CCI categorised like this? It seems to have ended up with about three quarters of the sample in the bottom category which hardly seems optimal. A glossary of terms might be a good idea too. I know it was spelled out earlier but it is awkward to have to look back to find it (for readers who print articles)”*

**Answer:** Thank you for the comment. The categories for Charlson Comorbidity Index were chosen to define low multimorbidity (up to 1 condition), mild morbidity (more than 1 condition, or one conditions with complications) and severe multimorbidity (more than 4 conditions). In order to address our research question, we needed a referent group with low multimorbidity. In our relatively healthy cohort, the low multimorbidity group formed the majority of the study population.

We have included this justification in our Methods.

CCI was classified in 3 groups: low morbidity CCI ≤1 (referent group), mild morbidity 2-3, and severe morbidity ≥4). These groups were chosen to discriminate between those with small comorbidity burden (up to 1 chronic condition), mild morbidity (2-3 chronic conditions) and severe morbidity (more than 4 chronic conditions).

We have included a glossary of terms.

**Comment:** *“Page 6 The paragraph starting ‘Sixty two percent’ is, strictly speaking part of the results.”*

**Answer:** The Reviewer is correct. We have deleted that information from Methods and added it to Results.

**Comment:** *“Page 6 Presumably ‘men are less likely . . . ’ applies to this condition. I do not think it is universal in health care.”*

**Answer:** It has been previously reported that men are less likely to be investigated or treated for osteoporosis. We have now provided a reference to support this claim.

**Comment: *“****Page 7*

*Given the substantial differences in fracture site between men and women I am surprised that it was not included as an adjustment variable in the models especially when sex differences are being investigated. For instance about 51% of the women but only 38% of the men sustained a distal fracture (using the DXA columns, the Rx columns have very different N). Indeed the results section shows the authors have noted this difference.”*

**Answer:** This is an important comment. Given the major differences between women and men, all our analyses were sex specific. In addition, we have conducted a separate analysis with women and men together to determine whether there is an interaction between sex and outcomes. From this additional analysis we have only reported the interaction.

We have made this clear in Methods:

Given the differences in fracture risk between women and men, all analyses conducted in this study were sex specific.

**Comment:** *“Page 7 If the cohort was arrived at by probability sampling as page 4 states then it needs to be taken into account in the analysis but I do not see it mentioned here or later in the results.”*

**Answer:** Thank you for the comment. The probability sampling refers only to the method of participants recruitment in the 45 and Up Study. Our study cohort included all participants with a confirmed index fracture and classified as high risk without further sampling.

**Comment**: *“Page 7 What exactly does the* *± notation mean? If this is a confidence interval then I am surprised that it is symmetrical given the proximity of the ages to the practical upper bound.”*

**Answer:** The ± sign was used in relation to SD. We have deleted it from Table 1 to avoid confusion.

**Comment*: “****Page 7 What does 27 − 53% mean? Is this a confidence interval and if so how was it calculated and what is the estimate about which it lies? If it is not a confidence interval then what is it?”*

**Answer:** The numbers quoted by the Reviewer represent the range of the magnitude of ORs for different levels of the Charlson Comorbidity Index for women and men. To avoid confusion, we have now also reported the adjusted ORs in parentheses.

The following changes were made to the manuscript.

Compared to low multimorbidity (CCI ≤ 1), mild multimorbidity (CCI between 2 and 3) and severe multimorbidity (CCI ≥ 4) were significantly associated with lower odds of DXA. The magnitude of association ranged between 27-53% lower for mild multimorbidity [adjusted OR, 0.73 (95% CI, 0.61 – 0.87) and 0.47 (95% CI, 0.33 – 0.68) for women and men, respectively] and between 48-57% lower for severe multimorbidity [adjusted OR, 0.43 (0.30 – 0.62) and 0.52 (0.31 – 0.84) for women and men, respectively].

**Comment:** *“Page 8 If there is a clear sex difference then we need to see the values for each sex separately and not just, as on the previous page, an overall value which applies to neither sex.”*

**Answer:** Thank you for the comment. Previously we have only reported the full OR and 95% CI for women and men in Tables only. In the revised manuscript, we have added this information in the Results section as well.

The following changes were added to the text.

Compared to low multimorbidity (CCI ≤ 1), mild multimorbidity (CCI between 2 and 3) and severe multimorbidity (CCI ≥ 4) were significantly associated with lower odds of DXA investigation. The magnitude of association ranged between 27-53% lower for mild multimorbidity [adjusted OR, 0.73 (95% CI, 0.61 – 0.87) and 0.47 (95% CI, 0.33 – 0.68) for women and men, respectively] and between 48-57% lower for severe multimorbidity [adjusted OR, 0.43 (0.30 – 0.62) and 0.52 (0.31 – 0.84) for women and men, respectively].

Similar to DXA investigation, mild multimorbidity (CCI between 2 and 3) and severe multimorbidity (CCI ≥ 4) were associated with lower odds of treatment. The magnitude of association ranged between 15 – 25% lower for mild multimorbidity [adjusted OR, 0.85 (95% CI, 0.74 – 0.98) and 0.75 (95% CI, 0.59 – 0.94), for women and men respectively] and between 22% - 63% lower for severe multimorbidity [adjusted OR, 0.78 (95% CI, 0.61 – 0.99) and 0.37 (95% CI, 0.23 – 0.53), for women and men, respectively].

**Comment: *“****Page 8 How were the figures for the numbers prevented arrived at and what model was used to determine the confidence intervals quoted here? This is not spelled out in the methods as far as I can see”*

**Answer:** Thank you for the comment. We have estimated the number of subsequent fractures possibly prevented as the difference between the observed number of subsequent fractures and the estimated number of subsequent fractures. The estimated number of subsequent fractures was derived as being 30% lower than the observed number based on the treatment effects documented from randomised controlled trials. The point estimate and its variation (95% confidence interval) were estimated under an assumption of Poisson distribution.

We have provided now in Methods a statement on how the prevented fractures were estimated.

We performed an exploratory analysis to determine the number of fractures potentially prevented by osteoporosis treatment. Based on findings of a meta-analysis evaluating effect of bisphosphonate treatment on fracture risk, we assumed that treatment would reduce fracture risk by 30%[6] [4] [23].

The follow-up time was calculated from the date of index fracture to the date of second any/hip fracture, or death or end of follow-up (April 2017), whichever came first. The observed rate of any second, and hip second fracture was calculated as number of events over 1000 person-years follow-up, assuming a Poisson distribution. The number of prevented fractures was then obtained by subtracting from the observed number the estimated number of any/hip subsequent fracture occurring with an incidence rate lower by 30% than the observed rate.

**Comment:** *“Page 10 What is BMD? If it is bone mineral density, as seems possible, how plausible is it to use weight as a surrogate for it?”*

**Answer:** Thank you for the comment. The Reviewer is right, BMD stands for bone mineral density, and this is made clear in the manuscript now. BMD and weight are highly correlated and the Garvan Fracture Risk Tool has an algorithm based on weight when BMD is unavailable (https://www.garvan.org.au/promotions/bone-fracture-risk/calculator/ ). Both models were externally validated and performed well in fracture risk prediction. However, the model with weight had ~10% lower predictive accuracy compared to the model with BMD. We have added this as a limitation.

The following statement was added to limitations.

We used weight for the calculation of Garvan subsequent fracture estimates, which has ~10% lower predictive accuracy than the model with bone mineral density (BMD)[7].

**Comment:** *“Summary*

*The whole article is rather short of detail especially on why the authors did what they did. The non–use of survey weights and the apparent disregard of fracture site need attention.”*

**Answer:** Thank you for the comment. The manuscript was revised substantially. The current version of the manuscript provides a detailed description on how questionnaires and linked data were used together to define multimorbidity and to estimate fracture risk. We have also added a sensitivity analysis including all hip and vertebral fractures, regardless of their 10- year fracture risk estimates. We have also presented in the Results section the complete HR and 95% CI for our primary aims.

**REVIEWER # 2**

**Comment***: “This is an interesting and timely paper as the treatment gap in patients at high risk of fracture is receiving much attention. The potential role of comorbidities in the investigation and treatment of these patients is of interest.”*

**Answer:** Thank you for the positive comments on our manuscript.

**Comment:** *“I would make the following comments:
a) I would suggest that the title is altered to say 'association' instead of 'contribution' as we can't ascribe causality.”*

**Answer:** We agree with the Reviewer and have changed the title to: Multimorbidity is associated with lower chance of osteoporosis investigation and treatment in high-risk fracture patients in Australia: a prospective cohort study

**Comment:** *“b) I am a little unclear why patients with fractures, including hip and/or vertebral fractures, with Garvan risk scores <20 were not included in the analysis as many if not most guidelines would recommend strong consideration of investigation and/or treatment in such individuals. Can this be clarified? Do data for this group exist?”*

**Answer:** Thank you for the comment. Our manuscript has focused on a group of patients with fractures who are recognised as being at high-risk for subsequent fractures and considered as candidates for future fracture risk prevention. However, we agree with the Reviewer that hip and vertebral fractures are universally accepted as being high risk groups and therefore we have added an additional analysis based on these fractures regardless of their 10-year Garvan Fracture Risk estimates.

We have added the following statement to the Methods:

We have performed three sensitivity analyses including: 1) group with 10-year Garvan Fracture Risk ≥ 40%, 2) all fractures, as well as hip and vertebral fractures regardless of the 10-year Garvan Fracture Risk and 3) investigation by DXA in all participants regardless of whether they received osteoporosis treatment.

…..

The second sensitivity analysis requested by a reviewer was performed because most clinical guidelines recommend osteoporosis investigation and treatment initiation following all hip and vertebral fracture. Given that comorbidities are associated with factors included in the Garvan Fracture Risk calculator, we also conducted an additional analysis in the “all fracture” group.

The following statement was added to Results:

**Sensitivity analysis regardless of 10-year Garvan Fracture Risk (Hip and vertebral fractures and all fractures)**

Of the total 25,280 participants (n=16,191 women and n=9089 men), 3479 (21%) women and 2581 (28%) men sustained a hip or vertebral index fracture. Of these, 1339 (38%) women and 372 (14%) men received prior osteoporosis treatment and were further excluded from this analysis.

Mild multimorbidity (CCI between 2 and 3) was significantly associated with lower odds for DXA investigation in both sexes [adjusted OR; 0.71 (95% CI, 0.56 - 0.91) and 0.59 (95%CI, 0.44 - 0.79) for women and men, respectively], and lower odds for treatment in women only [adjusted OR, 0.79 (95% CI, 0.62 - 0.99) and 1.08 (95% CI, 0.92 - 1.53) for women and men, respectively]. Severe multimorbidity (CCI ≥ 4) was associated with both lower odds for DXA [adjusted 0.29 (0.18 - 0.49) and 0.40 (0.25 - 0.63), for women and men, respectively] and treatment initiation [adjusted OR, 0.70 (95% CI, 0.48 - 1.00) and 0.46 (95% CI, 0.30 - 0.71) for women and men, respectively] for both sexes.

The association between comorbidities and DXA investigation and osteoporosis treatment was also observed when all participants were analysed together.

**Comment*:*** *“c) I am also a little unsure of the rational to look at DXA measurement ONLY in those who didn't subsequently get treatment - I can't see why this shouldn't be explored in all patients with perhaps the sub-group analyses as a sensitivity analysis.”*

**Answer:** Thank you for the comment. We have excluded the group who received treatment from DXA model, because clinicians could initiate fracture prevention treatment either based on previous DXA or on the presence of other fracture risk factors. For this reason, we have considered that fracture risk was correctly identified in this group.

However, we have performed the analysis per Reviewer’s suggestion and have added it to the manuscript as a sensitivity analysis.

We have added the following statement in Methods.

The third sensitivity analysis was performed as requested to determine whether the exclusion of the treated participants affected the main outcomes.

We have added the following statement in the Results.

3) **Association between DXA investigation and multimorbidity in all participants regardless of whether they received osteoporosis treatment**

There were in total 2527 (32%) DXA referrals in women and 341 (13%) in men. The predictors for DXA were similar to the main analysis (Table S4). Mild multimorbidity (CCI between 2 and 3) was associated with lower odds of DXA in both sexes [adjusted OR, 0.73 (95% CI, 0.64 - 0.84) and 0.46 (95% CI, 0.35 - 0.61) for women and men, respectively]. Similar findings were observed for severe multimorbidity (CCI ≥ 4) [adjusted OR, 0.51 (95% CI, 0.40 - 0.65) and 0.38 (95% CI, 0.25 - 0.57) for women and men, respectively].

We have added Table S4 to the supplemental document.

Table S4 Clinical risk factors associated with DXA investigation in all participants regardless of treatment initiation

|  | Women | | Men | |
| --- | --- | --- | --- | --- |
|  | Age-adjusted OR | Multivariable OR | Age-adjusted OR | Multivariable OR |
| Age + 5 years | 0.78 (0.76 - 0.80) | 0.79 (0.77 - 0.82) | 0.48 (0.41 - 0.55) | 0.47 (0.41 - 0.55) |
| Number of comorbidities |  |  |  |  |
| 0 | Reference | Reference | Reference | Reference |
| 1 and 2 | 1.05 (0.92 - 1.21) | 1.09 (0.94 - 1.25) | 1.14 (0.69 - 1.90) | 1.14 (0.67 - 1.92) |
| ≥ 3 | 1.09 (0.94 - 1.26) | 1.15 (0.99 - 1.33) | 1.01 (0.61 - 1.67) | 1.02 (0.67 - 1.92) |
| Number of prior hospitalisations |  |  |  |  |
| 0 | Reference | Reference | Reference | Reference |
| 1 and 2 | 0.78 (0.69 - 0.88) | 0.80 (0.71 - 0.91) | 0.58 (0.45 - 0.75) | 0.61 (0.47 - 0.79) |
| ≥3 | 0.46 (0.33 - 0.63) | 0.49 (0.35 - 0.67) | 0.41 (0.26 - 0.63) | 0.43 (0.27 - 0.67) |
| Charlson Comorbidity Index |  |  |  |  |
| 1 | Reference | Reference | Reference | Reference |
| 2 and 3 | 0.71 (0.62 - 0.81) | 0.73 (0.64 - 0.84) | 0.45 (0.35 - 0.59) | 0.46 (0.35 - 0.61) |
| ≥ 4 | 0.49 (0.38 - 0.62) | 0.51 (0.40 - 0.65) | 0.36 (0.24 - 0.54) | 0.38 (0.25 - 0.57) |
| Ischaemic heart disease | 1.34 (1.14 - 1.57) | 1.40 (1.17 - 1.68) | 1.42 (1.02 - 1.97) | 1.42 (1.01 - 1.98) |
| Arrhythmias | 0.75 (0.62 - 0.92) | 0.75 (0.60 - 0.93) | 0.77 (0.56 - 1.05) | 0.74 (0.54 - 1.03) |
| Stroke | 0.91 (0.70 - 1.18) | 0.94 (0.68 - 1.30) | 0.61 (0.39 - 0.97) | 0.63 (0.38 - 1.05) |
| Diabetes | 0.50 (0.37 - 0.68) | 0.57 (0.41 - 0.80) | 0.75 (0.42 - 1.34) | 0.74 (0.31 - 1.32) |
| Respiratory disease | 0.99 (0.88 - 1.12) | 1.08 (0.94 - 1.25) | 1.07 (0.79 - 1.46) | 1.15 (0.84 - 1.58) |
| Renal disease | 0.60 (0.40 - 0.90) | 0.58 (0.37 - 0.91) | 0.61 (0.34 - 1.09) | 0.56 (0.31 - 1.04) |
| Dementia | 0.20 (0.11 - 0.36) | 0.22 (0.12 - 0.39) | 0.31 (0.14 - 0.67) | 0.33 (0.14 - 0.76) |
| Cancer | 1.07 (0.95 - 1.21) | 1.17 (1.02 - 1.34) | 0.95 (0.74 - 1.21) | 0.91 (0.70 - 1.17) |
| Peptic ulcer | 1.14 (1.01 - 1.30) | 1.17 (1.01 - 1.35) | 0.79 (0.56 - 1.12) | 0.82 (0.58 - 1.17) |
| Aged care residency | 0.62 (0.44 - 0.86) | 0.57 (0.40 - 0.92) | 0.89 (0.42 - 1.88) | 0.96 (0.43 - 2.15) |
| Disability | 0.74 (0.62 - 0.89) | 0.76 (0.62 - 0.92) | 0.55 (0.35 - 0.87) | 0.63 (0.39 - 1.02) |
| Smoking | 0.71 (0.57 - 0.89) | 0.68 (0.54 - 0.86) | 0.58 (0.28 - 1.18) | 0.55 (0.25 - 1.17) |
| Private health insurance | 1.23 (1.11 - 1.36) | 1.33 (1.18 - 1.49) | 1.52 (1.20 - 1.93) | 1.49 (1.17 - 1.90) |
| Married | 1.12 (1.01 - 1.23) | 1.13 (1.01 - 1.26) | 0.83 (0.65 - 1.07) | 0.80 (0.62 - 1.04) |

**REVIEWER # 3**

**Q:** *“This manuscript by Bliuc et al is an original prospective study assessing the impact of comorbidities on DXA referral and treatment initiation in a population cohort after an index fracture, in patients at high risk of refracture and not already receiving anti-osteoporotic drugs.
They show that overall in this population the rate of DXA referral is only 17% in women 7% in men, and treatment initiation is 22% in women and 14% in males.
The higher the Charlson Comorbidity Index, the lower chance the patients are assessed for DXA or treated, in particular in men.
If these findings have been pointed out previously, there is little published evidence addressing specifically the interaction of various comorbidities on DXA screening and treatment initiation. This study by Bliuc et al clearly show that the the risk of fracture is either under-estimated or under prioritized in people with multiple chronic conditions.
The reasons for that finding cannot be explained here, but this fact should be reflected and considered by the medical community, whatever the specialty of the reader is. The prevention of osteoporotic fractures is largely suboptimal, in particular in patients at higher risk. In other diseases, such as cardiovascular diseases for example, such a finding would be unaccepted.
As an illustration, in Plos Medicine system, osteoporosis field of expertise is under Woman's health ! It should be under Endocrinology or/and Rheumatology!
It is robust and important for the medical community, way beyond bone health specialists.”*

**Answer:** Thank you for the positive comments. We are hoping that this work will raise fracture prevention awareness among clinicians.

**Comment***: “Main remarks:
- The inclusion criteria was based on prior fracture (index fracture), Gravan Fracture risk without DXA > 20% , meaning that it is mainly on falls and weight that patients were selected. How does the CCI correlate with Gravan Fracture?”*

**Answer:** Thank you for the comment. The Garvan Fracture Risk (GFR) calculation is based on age, sex, prior fracture, weight and falls. Therefore, in this cohort fracture risk was based on age, sex, falls, \ weight and also the number of prior fractures (up to 2 fractures prior to the index fracture). The high-risk group had significantly higher CCI then the lower risk group. For women, the distribution of CCI (≤ 1, 2 and 3, ≥ 4) was 70%, 24%, 7% for GFR ≥ 20%, and 88%, 9%, 3% for GFR <20% (p-value <0.0001). For men, CCI distribution ((≤ 1, 2 and 3, ≥ 4) was 47%, 38%, 15% for GFR ≥ 20%, and 75%, 18%, 7% for GFR <20% (p-value <0.0005).

We have now reported the differences in the Results.

Compared to the low-risk group, in the high-risk group, individuals were older [age, years mean ±SD, women: 77 ±10 vs 57±4 and men: 86 ± 5 vs 65 ± 8, for high vs low-risk groups, respectively], and had more severe fractures [index hip and vertebral fracture n (%), women: 3051 (26%) vs 223 (6%) and men: 1500 (45%) vs 981 (18%) for high vs low risk groups, respectively]. Those in the high-risk group also had a higher CCI [women, (≤ 1, 2 and 3, ≥ 4) was 70%, 24%, 7% for high-risk and 88%, 9%, 3% for low-risk (p-value <0.0001); men 47%, 38%, 15% for high, and 75%, 18%, 7% for low-risk (p-value <0.0005)] and a higher prevalence of cardio-vascular, diabetes, respiratory, renal disease, cancer and dementia. There was no difference in the level of education between the two fracture risk groups.

**Comment:** *“Why was the Gravan risk used to select the patients since they are already at higher risk of fracture as they have a fracture history? Would the results be the same in patients with Gravan Fracture < 20 %? Would the rate of patients in each category (DXA / Tt initiation) be similar ? Could dementia and diabetes be significantly associated with falls?””*

**Answer:** The Reviewer is correct that our entire fracture cohort is at high-risk of future fracture. We have chosen to report the high-risk group, instead of the whole cohort, because this group is universally accepted by clinical guidelines and medical practitioners as being candidates for osteoporosis investigation and treatment.

However, the Reviewer is correct that the variables included in the Garvan Fracture Risk calculator correlate with comorbidities. The risk of falling is indeed higher in individuals with diabetes and dementia. However, the published Garvan Fracture Risk calculator [https://www.garvan.org.au/promotions/bone-fracture-risk/calculator/] does not include comorbidities as predictor of fracture.

In the whole cohort, DXA investigation was slightly higher (24% for women and 9% for men) and the uptake of treatment was slightly lower (18% of women and 10% of men) than for the high-risk group. However, the predictors of DXA investigation and treatment were similar to the high-risk group. Importantly, CCI was a significantly associated with lower odds of both DXA investigation [CCI between 2 and 3, adjusted OR, 0.76 (95% CI, 0.68 - 0.85) and 0.84 (95% CI, 0.71 - 0.99) and CCI ≥ 4, adjusted OR, 0.56 (95% CI, 0.76 - 0.69) and 0.66 (95% CI, 0.51 - 0.99) for women and men, respectively] and treatment [adjusted ORs: CCI between 2 and 3, 0.87 (95% CI, 0.77 – 0.99) and 0.85 (0.73 – 0.99); CCI ≥ 4, 0.86 (95% CI, 0.69 – 0.99) and 0.70 (0.54 – 0.89), for women and men, respectively].

While we would like to keep the main aim focused on the high-risk group, we are happy to include the whole fracture cohort as a sensitivity analysis.

The following statement was included in the Methods:

**Sensitivity analyses**

We have performed three sensitivity analysis including: 1) group with 10-year Garvan Fracture Risk ≥ 40%, 2) all fractures, as well as hip and vertebral fractures regardless of the 10-year Garvan Fracture Risk and 3) investigation by DXA in all participants regardless of whether they received osteoporosis treatment.

The first sensitivity analysis was performed because clinical guidelines in some countries recommend osteoporosis treatment at a higher fracture risk threshold. The second sensitivity analysis requested by a reviewer was performed because most clinical guidelines recommend osteoporosis investigation and treatment initiation following all hip and vertebral fractures. Given that some comorbidities are associated with factors included in the Garvan Fracture Risk calculator, we conducted an additional analysis in the “all fracture” group. The third sensitivity analysis was performed as requested to determine whether the exclusion of the treated participants affected the main outcomes.

The following statement was added to the Results:

The association between comorbidities and DXA investigation and osteoporosis treatment was also observed when all participants were analysed together.

**Comment*:”*** *- Many non bone health specialists physicians have in mind that Calcium and vitamin D are effective anti-osteoporotic drugs reducing fracture risk. Did the authors search for calcium and vitamin D alone in the cohort?”*

**Answer**: Thank you for the comment. Unfortunately, we cannot ascertain the uptake of calcium and vitamin D alone in this cohort, as this information is not available in the prescription dataset because they are purchased without prescription.

We have added this as a limitation to our study.

We did not have prescription information on calcium and vitamin D, which may be considered by some medical practitioners as fracture prevention therapy.

**Comment*:”****- Regarding competing risk of mortality, would it be possible to show the data for males and females in each CCI group?”*

**Answer:** The Reviewer has raised an important issue. It is well known that CCI is a predictor of mortality risk in general population. However, in this fracture cohort we believe that mortality risk should not be isolated from the risk of subsequent fracture as fracture itself also predicts mortality. A cause-specific competing risk model is recommended for aetiology research to examine an association as it has been showed to be superior to other competing risk models, including the Fine-Gray subdistribution competing risk model[8, 9]. We have therefore conducted a cause-specific competing risk model for subsequent fracture risk, accounting for competing risk of death.

The following statement is added to Methods:

Given that multimorbidity is also associated with increased risk of mortality we have also performed a cause-specific competing risk model for subsequent fracture, accounting for competing risk of death.

We have added the following statement to Results.

**Association of multimorbidity with subsequent fracture and mortality risk**

Mild morbidity (CCI between 2 and 3) was associated with increased risk of subsequent fracture [HR, 1.18 (95% CI, 1.04 – 1.34) and 1.24 (95% CI, 1.06 – 1.44) for women and men, respectively] and increased risk of mortality [HR, 1.56 (95% CI, 1.26 – 1.92) and 1.53 (95% CI, 1.23 – 1.91), for women and men, respectively]. Severe multimorbidity (CCI ≥ 4) was also associated with increased risk of subsequent fracture [HR, 1.56 (95% CI, 1.26 – 1.92) and 1.53 (95% CI, 1.23 – 1.91) for women and men, respectively] and increased risk of mortality [HR, 5.16 (95% CI, 4.34 – 6.00) and 4.15 (95% CI, 3.68 – 4.69)].

**Comment:** *“- Discussion: could the authors speculate on the reasons for these findings? What would be the percentage of patients with a clear contra-indication of therapy?”*

**Answer:** Thank you for the comment. We believe there are very few people would have a strong contraindication for treatment. Such an example would be a person with no expectation of minimum recovery following a stroke. However, in this study we could not ascertain the exact number of patients with treatment contra-indication.

We have added this to our limitations.

Lastly, we could not ascertain in this study the number of participants with a clear contra-indication for treatment.

**Comment:** *“Minor remark:
Table 1: Please indicate what Rx / NoRx stands for (treatment initiation?)”*

**A:** Thank you for the comment. The Reviewer is correct. Rx stands for treatment initiation. We have made this clear in Table 1.

**References**

1.	Pratt NL, Kerr M, Barratt JD, Kemp-Casey A, Kalisch Ellett LM, Ramsay E, et al. The validity of the Rx-Risk Comorbidity Index using medicines mapped to the Anatomical Therapeutic Chemical (ATC) Classification System. BMJ Open. 2018;8(4):e021122.

2.	Thygesen SK, Christiansen CF, Christensen S, Lash TL, Sørensen HT. The predictive value of ICD-10 diagnostic coding used to assess Charlson comorbidity index conditions in the population-based Danish National Registry of Patients. BMC Medical Research Methodology. 2011;11(1):83.

3.	Lujic S, Simpson JM, Zwar N, Hosseinzadeh H, Jorm L. Multimorbidity in Australia: Comparing estimates derived using administrative data sources and survey data. PLoS ONE. 2017;12(8).

4.	Banks E, Redman S, Jorm L, Armstrong B, Bauman A, Beard J, et al. Cohort profile: the 45 and up study. Int J Epidemiol. 2008;37(5):941-7.

5.	45, Collaborators US. Cohort Profile: The 45 and Up Study. International Journal of Epidemiology. 2007;37(5):941-7.

6.	Byun J-H, Jang S, Lee S, Park S, Yoon HK, Yoon B-H, et al. The Efficacy of Bisphosphonates for Prevention of Osteoporotic Fracture: An Update Meta-analysis. J Bone Metab. 2017;24(1):37-49.

7.	Ahmed LA, Nguyen ND, Bjørnerem Å, Joakimsen RM, Jørgensen L, Størmer J, et al. External Validation of the Garvan Nomograms for Predicting Absolute Fracture Risk: The Tromsø Study. PLOS ONE. 2014;9(9):e107695.

8.	Austin PC, Lee DS, Fine JP. Introduction to the Analysis of Survival Data in the Presence of Competing Risks. Circulation. 2016;133(6):601-9.

9.	Lau B, Cole SR, Gange SJ. Competing Risk Regression Models for Epidemiologic Data. American Journal of Epidemiology. 2009;170(2):244-56.
